# ResectVol: A tool to automatically segment and characterize lacunas in brain images

**DOI:** 10.1002/epi4.12546

**Published:** 2021-10-12

**Authors:** Raphael F. Casseb, Brunno M. de Campos, Marcia Morita‐Sherman, Amr Morsi, Efstathios Kondylis, William E. Bingaman, Stephen E. Jones, Lara Jehi, Fernando Cendes

**Affiliations:** ^1^ Neuroimaging Laboratory Department of Neurology University of Campinas Campinas Brazil; ^2^ Epilepsy Center Cleveland Clinic Foundation Cleveland Ohio USA

**Keywords:** automatic segmentation, Epilepsy, MRI, surgical outcome, volumetry

## Abstract

**Objective:**

To assess and validate the performance of a new tool developed for segmenting and characterizing lacunas in postoperative MR images of epilepsy patients.

**Methods:**

A MATLAB‐based pipeline was implemented using SPM12 to produce the 3D mask of the surgical lacuna and estimate its volume. To validate its performance, we compared the manual and automatic lacuna segmentations obtained from 51 MRI scans of epilepsy patients who underwent temporal lobe resections.

**Results:**

The code is consolidated as a tool named *ResectVol*, which can be run via a graphical user interface or command line. The automatic and manual segmentation comparison resulted in a median Dice similarity coefficient of 0.77 (interquartile range: 0.71‐0.81).

**Significance:**

Epilepsy surgery is the treatment of choice for pharmacoresistant focal epilepsies, and despite the extensive literature on the subject, we still cannot predict surgical outcomes accurately. As the volume and location of the resected tissue are fundamentally relevant to this prediction, researchers commonly perform a manual segmentation of the lacuna, which presents human bias and does not provide detailed information about the structures removed. In this study, we introduce *ResectVol*, a user‐friendly, fully automatic tool to accomplish these tasks. This capability enables more advanced analytical techniques applied to surgical outcomes prediction, such as machine‐learning algorithms, by facilitating coregistration of the resected area and preoperative findings with other imaging modalities such as PET, SPECT, and functional MRI *ResectVol* is freely available at https://www.lniunicamp.com/resectvol.

**Abbreviations**
.png: Portable Network Graphics
CSF: cerebrospinal fluid
DSC: Dice similarity coefficient
FWHM: full width at half maximum
GM: gray matter
MNI: Montreal Neurological Institute
Postop‐MRI: postoperative MRI
Preop‐MRI: preoperative MRI
TLE: temporal lobe epilepsy
WM: white matter

**Key Points**

- Determining the resection volume of specific brain structures may add information about cognitive and seizure outcomes in epilepsy surgery
- ResectVol is a fully automatic tool that calculates the volume and segments the anatomic structures within the surgical lacuna using MRI scans
- The ResectVol 3D segmentation enables the comparison of resected structures and preoperative multimodal imaging
- This tool may help in developing more accurate predictive models for surgical outcome

## 1 INTRODUCTION

Epilepsy surgery is the treatment of choice for pharmacoresistant focal epilepsies.^1^ The current chances of achieving seizure freedom following epilepsy surgery are still highly variable, with the rates of complete postoperative seizure freedom ranging from 40% to 80%.^2^, ^3^, ^4^ Despite the extensive literature on the subject, we still cannot predict surgical outcomes accurately.^5^ The epilepsy surgery decision process is based on the analysis and concordance of multiple preoperative variables, such as EEG, MRI, and semiology. Some of these results are based on subjective impressions and imprecise measurements, making it challenging to develop accurate predictive models. Nevertheless, models using mainly clinical data were created with some success.^2^ The addition of findings from presurgical testing to the clinical data significantly enhanced the predictive discrimination of these models,^6^, ^7^ suggesting that a streamlined integration of data from multiple presurgical modalities can transform surgical outcome prediction. The development of new tools capable of precisely characterizing and differentiating the individual imaging features of each patient could be particularly helpful.

When evaluating epilepsy surgery outcomes, information on the amount and location of the resected tissue is undoubtedly important.^8^ However, in most outcomes research studies, patients are traditionally grouped into broad categories (eg, temporal lobe, frontal lobe, and posterior quadrant resections) to fit the inputs of traditional statistical methodologies. The downside is that by oversimplifying data on the surgical lacuna, we risk losing valuable predictive information on an individual patient basis. To overcome this limitation, researchers have used manual segmentation of the surgical lacuna as a quantitative instrument to better characterize the resection.^9^ Unfortunately, manual segmentation is time‐consuming and subject to variation depending on who performs it. Furthermore, available methods of automatic or semi‐automatic segmentation of the volume of the lacuna do not provide information on which brain anatomic regions were resected.^10^

In this study, we present and validate a tool developed by our group that automatically delineates and provides a 3D mask of the surgical lacuna, calculates the volume of the tissue resected, and identifies which brain structures were removed. This tool will facilitate and enhance our ability to evaluate surgical resections in detail and generate a range of possibilities to analyze the area resected and its relationship with other neuroimaging modalities and surgical outcomes. Our ultimate goal is to use this tool to improve our ability to predict postoperative seizure freedom.

## 2 METHODS

We developed *ResectVol*, a fully automatic, user‐friendly tool that obtains surgical lacuna segmentations and identifies the brain structures inside the lacuna using the pre‐ (Preop‐MRI) and postoperative MRI (Postop‐MRI) 3D T1‐weighted images. We validate its performance in a cohort of temporal lobe epilepsy patients who underwent surgery.

This study was conducted with approval from the Cleveland Clinical Foundation Institutional Review Board. Informed consent was waived due to the retrospective nature of the data collection. All images used in this study were anonymized.

### 2.1 Validation

To validate *ResectVol*, we used MRI datasets from patients with temporal lobe epilepsy (TLE) showing preoperative MRI signs of hippocampal sclerosis or a normal MRI, who underwent epilepsy surgery at the Cleveland Clinic Epilepsy Center from 2010 to 2019. We only included patients with available 3D T1‐weighted Preop‐MRI and Postop‐MRI. We randomly selected 60 individuals with temporal lobe epilepsy (TLE) who met inclusion criteria, which is approximately two times the number used in a similar study.^11^ After reviewing the quality of MR images, we excluded 4 cases due to MRI artifacts and 2 cases due to MRI signs of gliosis secondary to postoperative infection. In order to avoid the interference of blood and edema in the segmentation, we excluded patients whose postoperative MRI was performed less than five months after surgery (n = 3). Hence, 51 image datasets were used in the final analysis (24 men, 27 women; median age: 39.6 years; range: 12.4‐68.5).

### 2.2 Imaging protocol

The 3D T1‐weighted images were acquired across 1.5 and 3 T MRI scanners with different protocols to validate the segmentation tool in diverse conditions. Imaging parameters varied within the following ranges: voxel size = 0.39 × 0.39 × 0.8 to 1 × 1 × 1.5 mm³; TR = 8.5‐2300 ms; TE = 2.30‐4.92 ms; flip angle = 8‐25°; and image matrix = 192 × 192‐512 × 512. There were nine different MRI scanners (one by Philips and 8 by SIEMENS). A full description of imaging protocols is available in Table S1.

### 2.3 Automatic segmentation

We created ResectVol using MATLAB (version R2020b, The MathWorks, Inc) and SPM12^12^ (version 7771) to perform the automatic lacuna segmentation. A detailed description of the image processing pipeline can be found in Appendix S1. Briefly, the algorithm relies on the identification of brain tissues (gray matter (GM), white matter (WM), and cerebrospinal fluid (CSF)) in the Preop‐MRI and Postop‐MRI. Tissue maps are processed and then compared to identify the surgical resection volume. Figure S1 highlights the main steps in the pipeline.

In summary, six main results are generated: (a) the lacuna mask in the Preop‐MRI space and (b) in the MNI IXI549 standard space^13^; (c) the description text file with the lacuna volume and the resected volumes of the brain structures removed in the surgery; (d) the same file with the volumetric information in the MNI IXI549 space; (e) individual files with the resected portion masks for each brain structure removed in the surgery; and (f) an image file (.png format) showing axial slices of the segmentation overlayed onto the brain‐extracted Postop‐MRI. All 3D image files are saved in the Neuroimaging Informatics Technology Initiative (.nii) format.

### 2.4 Manual segmentation

Two epilepsy neurosurgery trainees supervised by the Cleveland Clinic epilepsy neurosurgeon W.B (rater 1 and rater 2) manually segmented the anatomical images using MRIcron^14^ (version 1.0.20190902) to draw the resected volume. All Postop‐MRIs were randomly split into two groups, and each was assigned to one of the raters.

To enable the comparison of the manual segmentation with the automatic one, the masks created by raters were registered into the space of the reference image (Preop‐MRI) since *ResectVol* masks were produced in the Preop‐MRI space. Finally, these images are lightly smoothed (FWHM = 1 × 1 × 1 mm^3^) and binarized (threshold = 0.5) to create the final manual masks used in the statistical comparisons.

### 2.5 Statistical analysis

All reported metrics are formatted as median (Q1‐Q3: 1st quartile–3rd quartile; range: minimum value–maximum value).

To assess the performance of *ResectVol*, we compared the lacuna masks and volume measurements generated by the manual and the automatic approaches. Manual masks were chosen as the gold standard. We calculated Pearson's correlation coefficient (r), the relative differences for the volume values, and the Dice similarity coefficient (DSC)^15^ for the lacuna masks. DSC ranges from 0 to 1 and is mathematically defined by the following equation:

$$\text{DSC =}\;\frac{2\times (M\cap A)}{M\cup A}$$

where M and A are the manual and automatic binary masks, respectively. A DSC =0 corresponds to no overlap between the manual and the automatic masks, and a DSC =1 means a perfect match.

## 3 RESULTS

### 3.1 Surgical outcomes

The median time interval between surgery and Postop‐MRI was 6 months (Q1‐Q3: 6‐6; range: 5‐96). Regarding surgical outcomes, 47% (24/51) of the participants were seizure free at the last follow‐up. The median postoperative follow‐up time was 51.58 months (Q1‐Q3: 28‐70; range: 7‐106).

### 3.2 Segmentation results

Automatic segmentation was conducted in a Microsoft Windows 10 Pro computer (version 10.0.19041) with 32 GB of RAM and an Intel Core i7‐8700K CPU at 3.7 GHz. The median processing time was 17.9 (Q1‐Q3: 11.5‐38.2; range: 7.8‐39.3) minutes.

The manual segmentation took approximately 30 minutes on average. It must be noted that manual segmentation consists exclusively of drawing the lacuna itself; that is, it does not provide the labeling and detailing of the individual brain structures inside it, as is done by *ResectVol*.

### 3.3 Performance assessment

**FIGURE 1:**
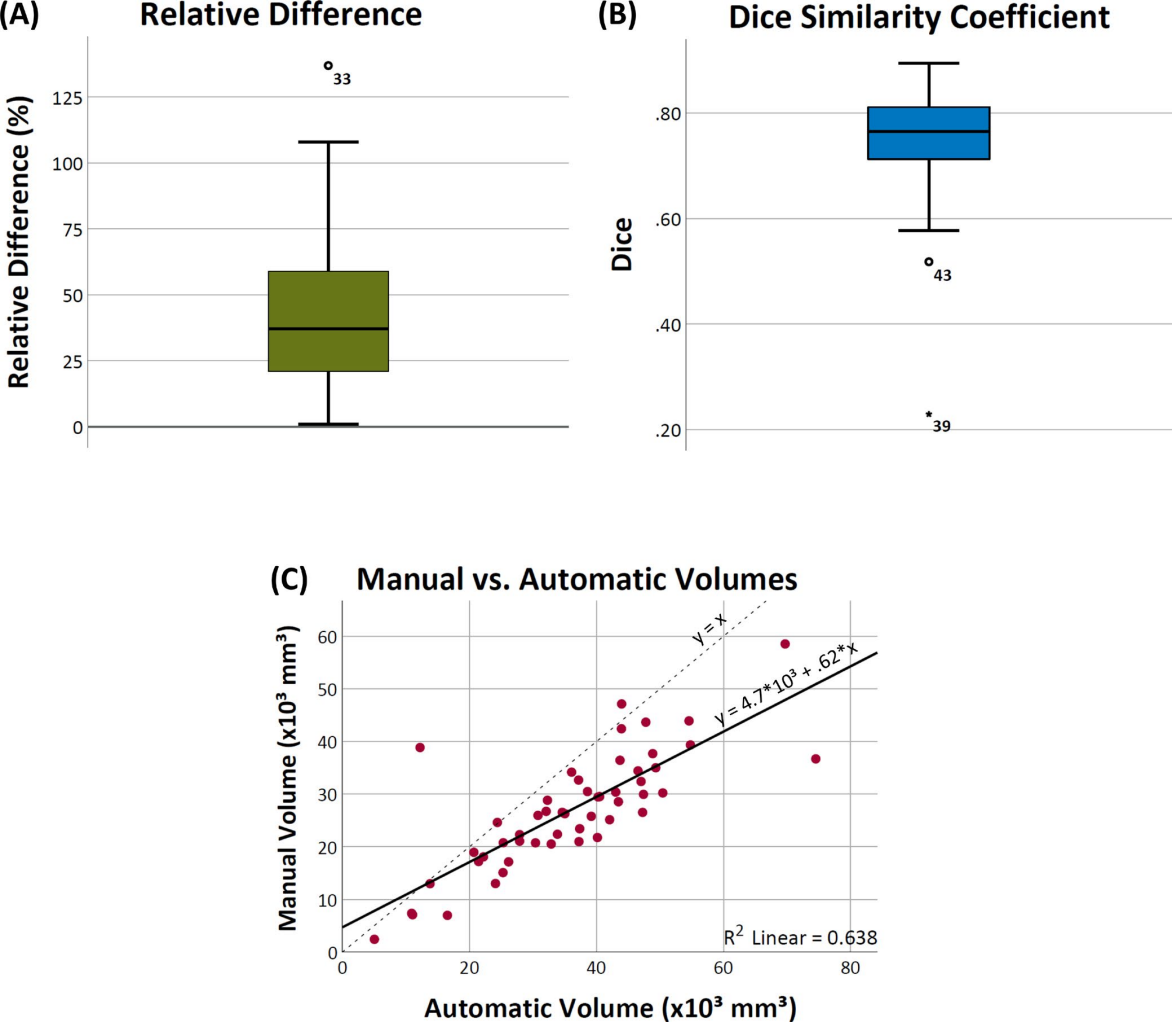
Performance metrics. Box plots for the (A) relative difference between approaches and (B) for the Dice coefficient, and (C) the scatter plot of the volumetric measurements with the linear fit (solid line; coefficient of determination (R²) = 0.638) and the reference line (dashed)

The median relative difference (Figure 1A) and the median DSC (Figure 1B) were 37.1 (Q1‐Q3: 21.0‐58.8; range: 0.9‐136.9) % and 0.77 (Q1‐Q3: 0.71‐0.81; range: 0.23‐0.89), respectively. The linear correlation between the automatic and the manual volumes obtained for the lacunas was r(49) = 0.8, *P* < .001 (Figure 1C).

**FIGURE 2:**
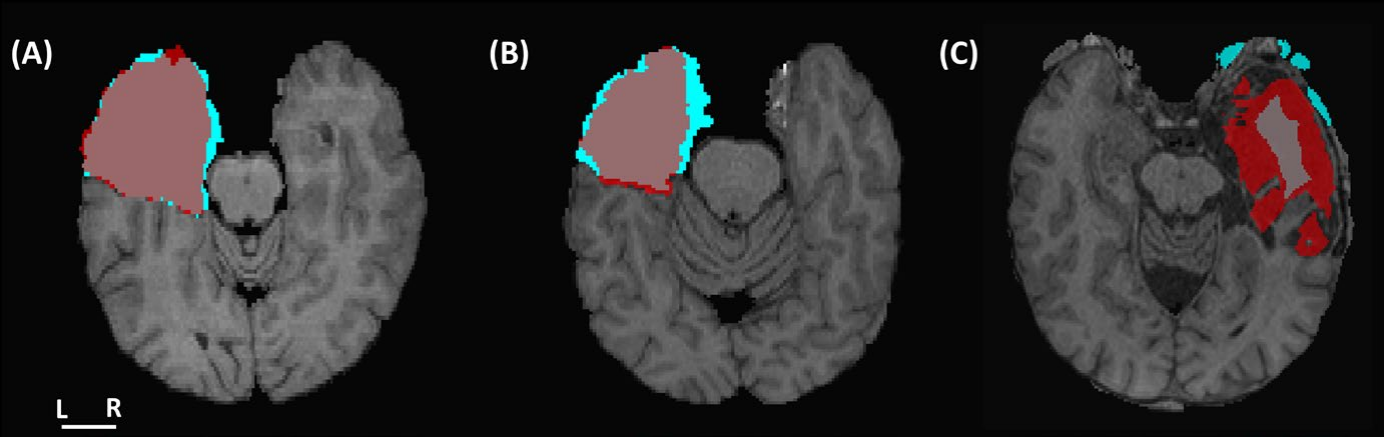
Segmentation examples. Manual (red) and automatic (cyan) lacuna masks overlayed onto the postoperative images. The images represent the (A) best (Dice similarity coefficient (DSC) = 0.89), (B) median (DSC = 0.76), and (C) worst (DSC = 0.23) DSCs obtained by the comparison of the manual and the automatic segmentation approaches

To offer a more visual perspective of the results, we display in Figure 2 the images and lacuna masks corresponding to the best (Figure 2A), median (Figure 2B), and worst (Figure 2C) DSC values. The poor segmentation in Figure 2C is likely a skull stripping error, which may be improved by adjusting the preprocessing parameters.

**FIGURE 3:**
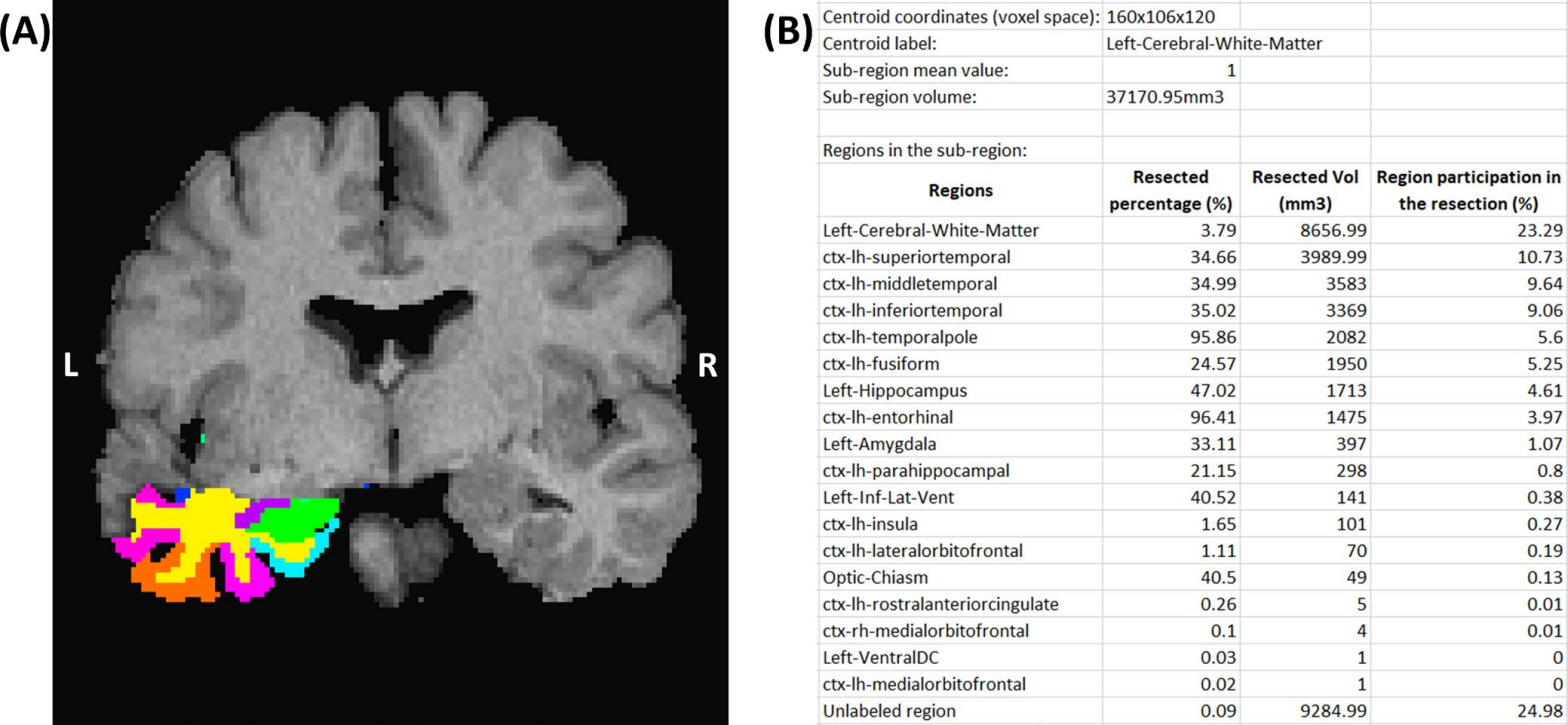
Characterization examples. (A) Brain structure masks overlayed onto the postoperative image and (B) the corresponding description file

Finally, an example of the labeled structures identified inside the lacuna and the associated description text file is given in Figure 3.

## 4 DISCUSSION

The segmentation of brain structures and lesions is a quantitative procedure frequently used in research and the clinical setting. Its importance lies in aiding surgical planning, defining treatment, and estimating progression and prognostics. However, manual segmentation of surgical lacunas is a time‐consuming task that only provides the volume of the resected tissue.

Here, we present and validate *ResectVol*, a user‐friendly and fully automatic tool for segmentation and characterization of surgical lacunas in postoperative MRIs. We compared the software's performance with the manual segmentation of postoperative MRIs of TLE surgeries with good results.

Although many studies have focused on segmenting brain lesions,^10^, ^16^ to the best of our knowledge, not as many have specifically targeted brain lacunas. Gau and colleagues^11^ claim to be the first group to have done so. They compared a fully automatic and a semi‐automatic approach against the manual segmentation masks and found a median DSC of 0.58 and 0.78, respectively. Their semi‐automatic method requires the user to click inside the lacuna and decide when the evolving contour has adequately identified its boundaries. In our study, using a fully automatic approach in a group of TLE patients, we obtained a median DSC = 0.77.

Along with the lacuna mask, *ResectVol* also saves the resected part of the brain structures inside the lacuna as individual masks and their volumes using the labeling definition from the Desikan atlas,^17^ since it is one of the most popular atlases available. It is possible to include other atlases in *ResectVol* to accommodate different types of studies. The volumes of the structures inside the lacuna will depend on the atlas chosen, but not the total volume of the lacuna.

We could not find any free tool that would accomplish a similar task without the need for additional intervention. The structure labeling is arguably the most advantageous feature in *ResectVol*. The individual masks of the resected brain structures enable researchers to use them as regions of interest for further analyses like tractography and functional connectivity. In addition, the resected volumes of these structures may support correlational studies to investigate their relationship with clinical scores allowing prediction and prognostication. It also raises the possibility to investigate the neuropsychological outcomes after surgical intervention. In future, we hope that improvements on this type of information can contribute to the development of sophisticated statistical models capable of accurately predicting surgical outcomes from simulated 3D resections.

**FIGURE 4:**
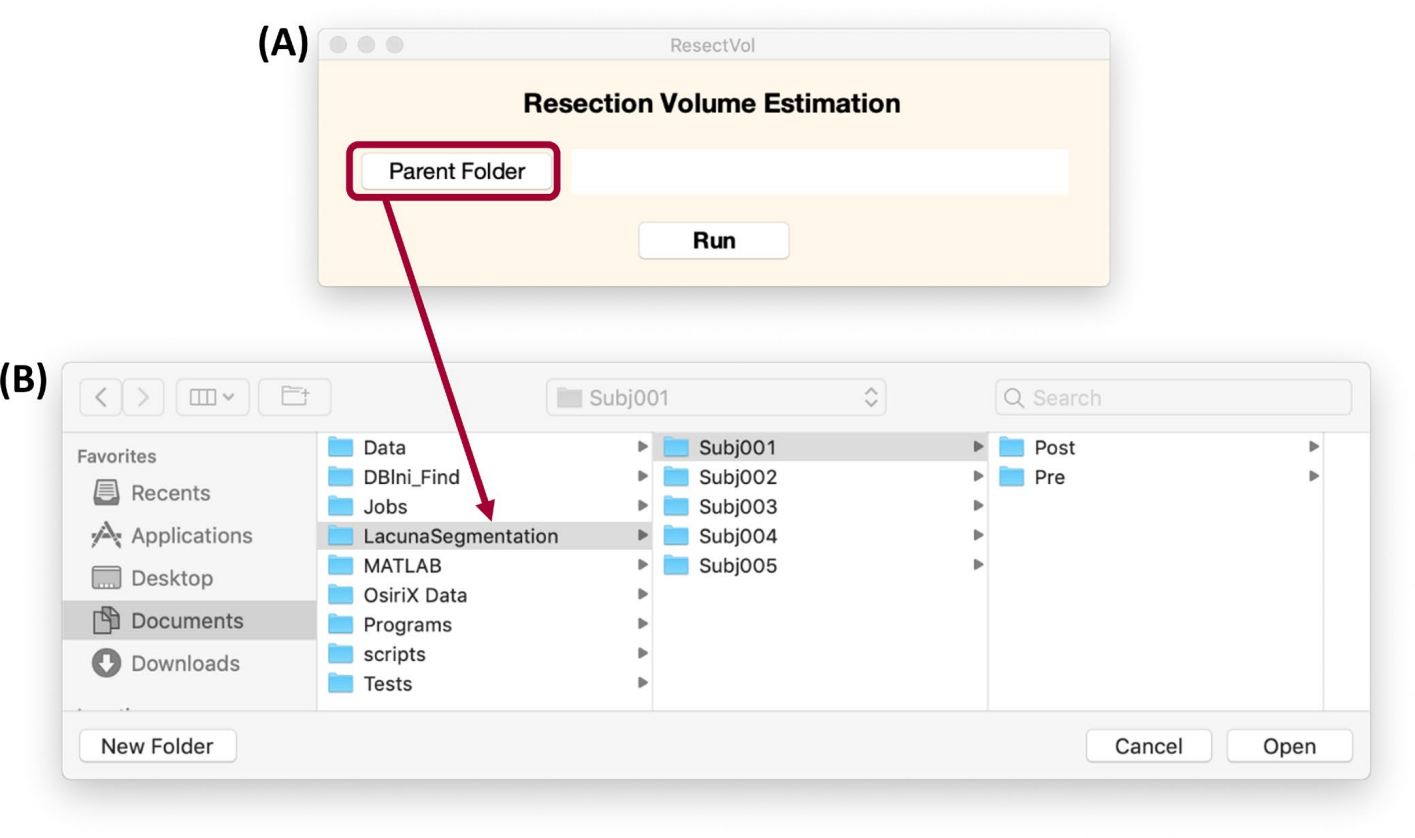
User interface. (A) Graphical user interface and (B) the selection of the parent folder containing all subjects' directories

*ResectVol* is freely available from https://www.lniunicamp.com/resectvol. Users can process one or multiple subject's datasets either through the graphical interface (Figure 4) or via command line.

### 4.1 Limitations

One limitation of our study was that we excluded patients whose postoperative MRI was performed less than 5 months after surgery. We based our decision on some preliminary tests and on the fact that sometimes it was a challenge to perform the manual segmentation in the presence of blood, gliosis, and edema. Depending on the number of perioperative changes in the acute postoperative MRI, it is still possible to use ResectVol; however, we strongly encourage the visual inspection of the results in this scenario and whenever the tool is used.

Our algorithm systematically produces slightly larger estimates, as demonstrated in Figure 2. This overestimation was intentional because when testing different binarization thresholds, we noted that more stringent values would generally find the right boundaries in some parts but underestimate others. Thus, a more liberal threshold would allow for a complete detection with the side effect of including nonlacuna voxels (especially CSF). As the accurate determination of the brain structures inside the lacuna (Figure 3A) was one of the main goals of this tool, and it was not affected by this overestimation, we decided to set more liberal thresholds as default. Nevertheless, as ResectVol is an open code software, users are entitled to change these values. Finally, although already mentioned, we must highlight that ResectVol relies on the contrast between tissues to obtain accurate results. Therefore, low contrast images that present the lacuna voxels (mainly CSF) with a brightness similar to brain tissue (GM and WM) will probably yield poor results. That is the case for the outliers in Figure 1A and B.

## 5 CONCLUSION

In this study, we assessed the performance of *ResectVol*, a tool capable of segmenting brain lacunas. Its validation was established in a cohort of patients with TLE who underwent surgical treatment, yielding a DSC larger than a previous study with the same intent (0.77 vs 0.58). These results indicate that ResectVol is an accurate tool that can be very helpful in large cohort studies due to its fully automatic nature, with the benefit of avoiding human bias in the segmentation process, besides providing informative details about the resected regions.

## DISCLOSURE

None of the authors has any conflict of interest to disclose.

## Supporting information

Fig S1

Table S1

Appendix S1

Supplementary Material
