# Supplementary figures and images for: ResectVol: A tool to automatically segment and characterize lacunas in brain images
Source: Epilepsia Open. 2021 Oct 12;6(4):720–6. doi: 10.1002/epi4.12546 (PMC8633465; doi:10.1002/epi4.12546)

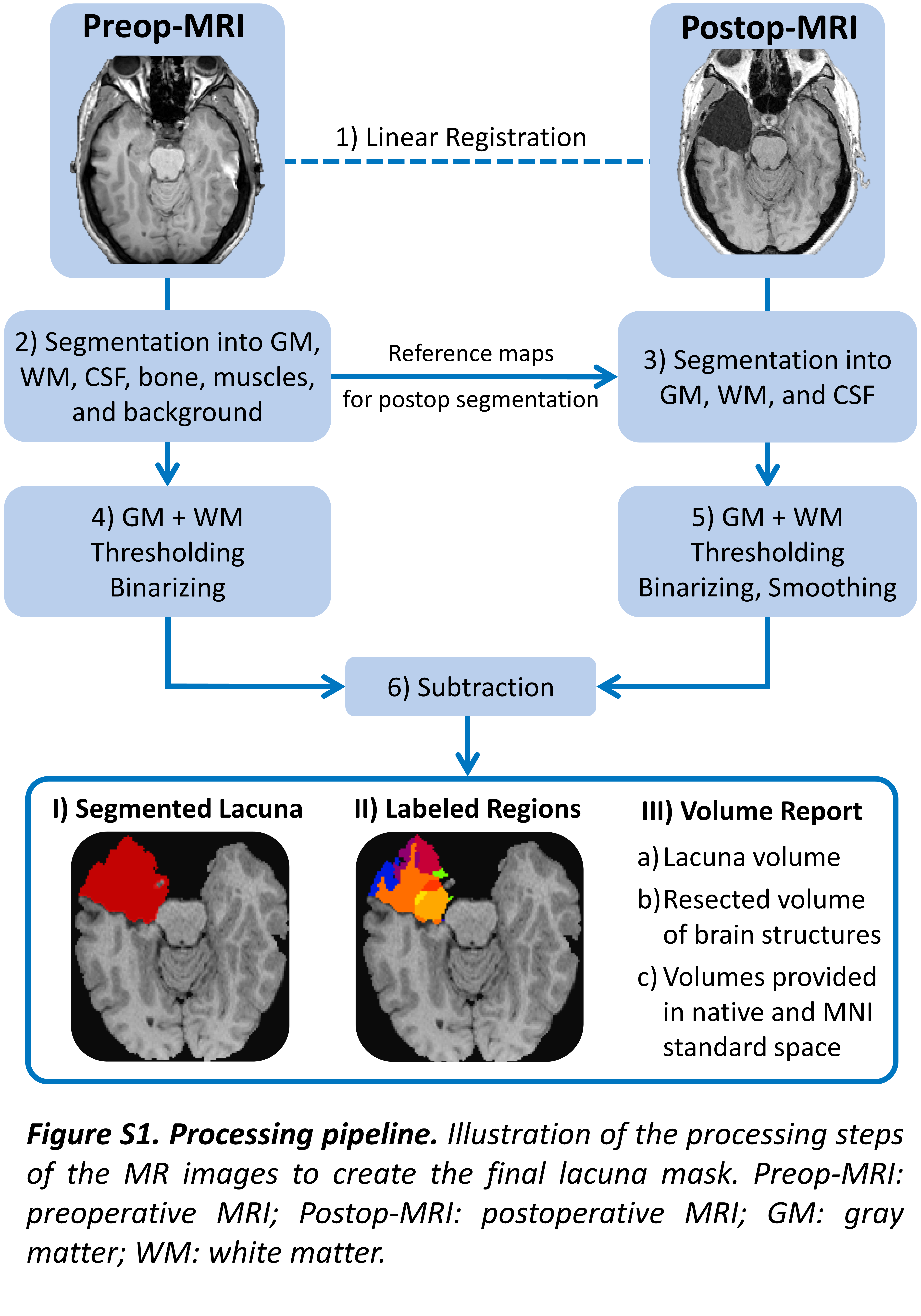

Supplement: Supplementary file 1 — Fig S1 [file EPI4-6-720-s001.tif]
