# Supplementary material for: ResectVol: A tool to automatically segment and characterize lacunas in brain images
Source: Epilepsia Open. 2021 Oct 12;6(4):720–6. doi: 10.1002/epi4.12546 (PMC8633465; doi:10.1002/epi4.12546)
Supplement: Supplementary file 2 — Table S1 [file EPI4-6-720-s004.pdf]

Table S1. Imaging parameters

Subj	Image	Matrix		Voxel Size		Slc. Thick.	Slices	Mag. Field	TR	TE	Flip Angle	Software	Manufacturer/Model
1	Preop	512	512	0.41	0.41	1	60	3	1800	2.56	10	syngo MR E11	SIEMENS/Skyra
1	Postop	512	512	0.41	0.41	1	157	3	1800	2.56	10	syngo MR E11	SIEMENS/Skyra
2	Preop	256	256	0.94	0.94	0.94	136	3	1860	3.40	10	syngo MR B15	SIEMENS/TrioTim
2	Postop	256	256	0.90	0.90	1.25	74	1.5	11	4.60	20	syngo MR B17	SIEMENS/Avanto
3	Preop	256	256	0.90	0.90	1.25	192	1.5	11	4.60	20	syngo MR B19	SIEMENS/Espree
3	Postop	256	256	1.00	1.00	1	78	3	10	4.45	8	syngo MR D13D	SIEMENS/Prisma_fit
4	Preop	256	256	1.00	1.00	1.2	22	3	2300	2.98	9	syngo MR E11	SIEMENS/Prisma_fit
4	Postop	256	256	0.90	0.90	1.25	60	1.5	11	4.60	20	syngo MR B19	SIEMENS/Avanto
5	Preop	256	256	1.00	1.00	1	83	3	10	2.30	8	2,6,3\2,6,3,4	Philips /Achieva
5	Postop	224	256	0.90	0.90	1.25	66	1.5	11	4.60	20	syngo MR B17	SIEMENS/Avanto
6	Preop	192	192	0.94	0.94	0.8	75	3	1800	3.01	9	syngo MR B15	SIEMENS/TrioTim
6	Postop	256	256	0.82	0.82	1	258	3	1860	3.40	10	syngo MR B17	SIEMENS/TrioTim
7	Preop	256	256	0.94	0.94	0.94	122	3	1860	3.40	10	syngo MR B15	SIEMENS/TrioTim
7	Postop	256	256	0.82	0.82	1	267	3	1800	2.40	10	syngo MR B19	SIEMENS/TrioTim
8	Preop	224	256	0.90	0.90	1.25	94	1.5	11	4.60	20	syngo MR B15	SIEMENS/Avanto
8	Postop	256	256	1.00	1.00	1	124	3	8	3.90	8	2,6,3\2,6,3,7	Philips /Achieva
9	Preop	512	512	0.47	0.47	0.94	1	3	1900	2.57	10	syngo MR D11	SIEMENS/Skyra
9	Postop	384	512	0.41	0.41	2	79	1.5	1990	4.38	10	syngo MR A35	SIEMENS/Symphony
10	Preop	384	384	0.39	0.39	0.8	110	3	1800	2.74	10	syngo MR D11	SIEMENS/Skyra
10	Postop	256	256	0.90	0.90	1.25	118	1.5	11	4.60	20	syngo MR B17	SIEMENS/Avanto
11	Preop	256	256	0.94	0.94	0.94	2	3	1860	3.40	10	syngo MR B15	SIEMENS/TrioTim
11	Postop	256	256	0.82	0.82	1	154	3	1860	3.40	10	syngo MR B17	SIEMENS/TrioTim
12	Preop	256	256	0.90	0.90	1.25	94	1.5	11	4.60	20	syngo MR B17	SIEMENS/Espree
12	Postop	512	512	0.41	0.41	1	167	3	1800	2.56	10	syngo MR D11	SIEMENS/Skyra
13	Preop	256	256	0.82	0.82	1	190	3	1800	2.40	10	syngo MR B17	SIEMENS/TrioTim
13	Postop	256	256	0.90	0.90	1.25	71	1.5	11	4.60	20	syngo MR B17	SIEMENS/Avanto
14	Preop	512	512	0.41	0.41	1	158	3	1800	2.56	10	syngo MR D11	SIEMENS/Skyra
14	Postop	512	512	0.41	0.41	1	256	3	1800	2.56	10	syngo MR D11	SIEMENS/Skyra
15	Preop	256	256	0.82	0.82	1	227	3	1800	2.40	10	syngo MR B17	SIEMENS/TrioTim
15	Postop	256	256	0.82	0.82	1.25	106	1.5	1990	4.38	10	syngo MR A30	SIEMENS/Symphony
16	Preop	256	256	1.00	1.00	1	150	3	8	3.90	8	2,6,3\2,6,3,7	Philips /Achieva
16	Postop	224	256	0.90	0.90	1.25	192	1.5	11	4.92	25	syngo MR D11	SIEMENS/Aera
17	Preop	512	512	0.41	0.41	1	158	3	1800	2.56	10	syngo MR D11	SIEMENS/Skyra
17	Postop	256	256	0.90	0.90	1.25	192	1.5	11	4.60	20	syngo MR B17	SIEMENS/Espree
18	Preop	256	256	0.90	0.90	1.25	99	1.5	11	4.60	20	syngo MR B17	SIEMENS/Avanto
18	Postop	256	256	0.90	0.90	1.25	192	1.5	11	4.60	20	syngo MR B17	SIEMENS/Avanto
19	Preop	224	256	0.90	0.90	1.5	92	1.5	11	4.60	20	syngo MR B17	SIEMENS/Avanto
19	Postop	256	256	0.78	0.78	1.25	176	1.5	1990	4.38	10	syngo MR A35	SIEMENS/Symphony
20	Preop	512	512	0.41	0.41	1	99	3	1800	2.56	10	syngo MR D11	SIEMENS/Skyra
20	Postop	256	256	0.90	0.90	1.25	120	1.5	11	4.60	20	syngo MR B19	SIEMENS/Espree
21	Preop	512	512	0.47	0.47	0.94	80	3	1900	2.57	10	syngo MR D11	SIEMENS/Skyra
21	Postop	512	512	0.41	0.41	1	256	3	1800	2.56	10	syngo MR D13	SIEMENS/Skyra
22	Preop	512	512	0.41	0.41	1	158	3	1800	2.56	10	syngo MR D13	SIEMENS/Skyra
22	Postop	224	256	0.90	0.90	1.25	160	1.5	1550	3.02	25	syngo MR B17	SIEMENS/Espree

23	Preop	512	512	0.41	0.41	1	143	3	1800	2.56	10	syngo MR D13	SIEMENS/Skyra
23	Postop	224	256	0.90	0.90	1.25	100	1.5	11	4.92	25	syngo MR D13	SIEMENS/Aera
24	Preop	512	512	0.41	0.41	1	94	3	1800	2.56	10	syngo MR D13	SIEMENS/Skyra
24	Postop	512	512	0.41	0.41	1	81	3	1800	2.56	10	syngo MR D13	SIEMENS/Skyra
25	Preop	256	256	0.82	0.82	1	145	3	1800	2.40	10	syngo MR B17	SIEMENS/TrioTim
25	Postop	512	512	0.41	0.41	1	6	3	1800	2.56	10	syngo MR D13	SIEMENS/Skyra
26	Preop	232	256	0.90	0.90	1.25	62	1.5	11	4.60	20	syngo MR B17	SIEMENS/Espree
26	Postop	512	512	0.41	0.41	1	256	3	1800	2.56	10	syngo MR D13	SIEMENS/Skyra
27	Preop	256	256	1.00	1.00	1	99	1.5	11	4.60	20	syngo MR B17	SIEMENS/Avanto
27	Postop	224	256	0.90	0.90	1.5	156	1.5	11	4.60	20	syngo MR B17	SIEMENS/Avanto
28	Preop	512	512	0.41	0.41	1	99	3	1800	2.56	10	syngo MR D11	SIEMENS/Skyra
28	Postop	512	512	0.41	0.41	1	243	3	1800	2.56	10	syngo MR D13D	SIEMENS/Prisma_fit
29	Preop	256	256	0.94	0.94	0.94	78	3	1860	3.40	10	syngo MR B17	SIEMENS/TrioTim
29	Postop	256	256	0.82	0.82	1	97	3	1800	2.69	10	syngo MR B20P	SIEMENS/Biograph
30	Preop	512	512	0.41	0.41	1	158	3	1800	2.56	10	syngo MR D13	SIEMENS/Skyra
30	Postop	256	256	0.90	0.90	1.25	192	1.5	11	4.60	20	syngo MR B17	SIEMENS/Espree
31	Preop	512	512	0.41	0.41	1	145	3	1800	2.56	10	syngo MR D13	SIEMENS/Skyra
31	Postop	512	512	0.41	0.41	1	256	3	1800	2.56	10	syngo MR D13D	SIEMENS/Prisma_fit
32	Preop	512	512	0.41	0.41	1	99	3	1800	2.56	10	syngo MR D13	SIEMENS/Skyra
32	Postop	512	512	0.41	0.41	1	256	3	1800	2.56	10	syngo MR D13	SIEMENS/Skyra
33	Preop	512	512	0.41	0.41	1	120	3	1800	2.56	10	syngo MR D13	SIEMENS/Skyra
33	Postop	512	512	0.41	0.41	1	187	3	1800	2.56	10	syngo MR D13	SIEMENS/Skyra
34	Preop	512	512	0.41	0.41	1	99	3	1800	2.56	10	syngo MR D13	SIEMENS/Skyra
34	Postop	216	256	1.00	1.00	1.25	192	1.5	11	4.60	20	syngo MR B19	SIEMENS/Avanto
35	Preop	512	512	0.41	0.41	1	197	3	1800	2.56	10	syngo MR D13	SIEMENS/Skyra
35	Postop	512	512	0.41	0.41	1	233	3	1800	2.56	10	syngo MR D13	SIEMENS/Skyra
36	Preop	256	256	0.94	0.94	0.94	78	3	1860	3.40	10	syngo MR B17	SIEMENS/TrioTim
36	Postop	512	512	0.41	0.41	1	136	3	1800	2.56	10	syngo MR D13	SIEMENS/Skyra
37	Preop	512	512	0.41	0.41	1	97	3	1800	2.56	10	syngo MR D13	SIEMENS/Skyra
37	Postop	512	512	0.41	0.41	1	162	3	1800	2.56	10	syngo MR E11	SIEMENS/Skyra
38	Preop	224	256	0.90	0.90	1.5	62	1.5	11	4.60	20	syngo MR B17	SIEMENS/Avanto
38	Postop	256	256	0.90	0.90	1.25	136	1.5	11	4.60	20	syngo MR B19	SIEMENS/Avanto
39	Preop	256	256	0.82	0.82	1	160	3	1800	2.69	10	syngo MR B20P	SIEMENS/Biograph
39	Postop	224	256	0.90	0.90	1.25	147	1.5	11	4.92	25	syngo MR E11	SIEMENS/Aera
40	Preop	256	256	0.82	0.82	1	43	3	1800	2.69	10	syngo MR B20P	SIEMENS/Biograph
40	Postop	512	512	0.41	0.41	1	71	3	1800	2.56	10	syngo MR D13	SIEMENS/Skyra
41	Preop	512	512	0.47	0.47	0.94	73	3	1900	2.57	10	syngo MR D13	SIEMENS/Skyra
41	Postop	224	256	0.90	0.90	1.25	158	1.5	11	4.92	25	syngo MR E11	SIEMENS/Aera
42	Preop	512	512	0.41	0.41	1	117	3	1800	2.56	10	syngo MR E11	SIEMENS/Skyra
42	Postop	512	512	0.41	0.41	1	251	3	1800	2.56	10	syngo MR E11	SIEMENS/Skyra
43	Preop	512	512	0.41	0.41	1	97	3	1800	2.56	10	syngo MR D13	SIEMENS/Skyra
43	Postop	512	512	0.41	0.41	1	195	3	1800	2.56	10	syngo MR E11	SIEMENS/Skyra
44	Preop	256	256	0.90	0.90	1.25	8	1.5	11	4.60	20	syngo MR B17	SIEMENS/Avanto
44	Postop	512	512	0.41	0.41	1	175	3	1800	2.56	10	syngo MR E11	SIEMENS/Skyra
45	Preop	184	256	0.98	0.98	1	78	1.5	1600	3.02	15	syngo MR B17	SIEMENS/Espree
45	Postop	512	512	0.41	0.41	1	210	3	1800	2.56	10	syngo MR E11	SIEMENS/Skyra

46	Preop	256	256	0.90	0.90	1.25	99	1.5	11	4.60	20	syngo MR B19	SIEMENS/Avanto
46	Postop	256	256	1.00	1.00	1.5	167	3	11	4.68	25	syngo MR E11	SIEMENS/Skyra
47	Preop	512	512	0.41	0.41	1	192	3	1800	2.56	10	syngo MR D13	SIEMENS/Skyra
47	Postop	512	512	0.41	0.41	1	63	3	1800	2.56	10	syngo MR E11	SIEMENS/Skyra
48	Preop	512	512	0.47	0.47	0.94	133	3	1900	2.57	10	syngo MR D13	SIEMENS/Skyra
48	Postop	512	512	0.41	0.41	1	157	3	1800	2.56	10	syngo MR E11	SIEMENS/Skyra
49	Preop	512	512	0.41	0.41	1	134	3	1800	2.56	10	syngo MR D13	SIEMENS/Skyra
49	Postop	512	512	0.41	0.41	1	144	3	1800	2.56	10	syngo MR E11	SIEMENS/Skyra
50	Preop	256	256	0.90	0.90	1.25	42	1.5	11	4.60	20	syngo MR B19	SIEMENS/Avanto
50	Postop	256	256	0.90	0.90	1.25	26	1.5	11	4.60	20	syngo MR B19	SIEMENS/Avanto
51	Preop	512	512	0.41	0.41	1	165	3	1800	2.56	10	syngo MR E11	SIEMENS/Skyra
51	Postop	512	512	0.41	0.41	1	89	3	1800	2.56	10	syngo MR E11	SIEMENS/Skyra
