# Supplementary material for: ResectVol: A tool to automatically segment and characterize lacunas in brain images
Source: Epilepsia Open. 2021 Oct 12;6(4):720–6. doi: 10.1002/epi4.12546 (PMC8633465; doi:10.1002/epi4.12546)
Supplement: Supplementary file 3 — Appendix S1 [file EPI4-6-720-s003.pdf]

Appendix S1 – ResectVol development

All steps are implemented in Matlab (version R2020b, The MathWorks, Inc., Natick, Massachusetts, United States) using SPM12[1] (version 7771) and are performed independently for each subject. In the first step, the Postop-MRI is linearly registered to the Preop-MRI, using a rigid-body model (based on Collignon et al.[2]) and optimizing the normalized mutual information as the cost function. In this step, the Postop-MRI is also resampled using a 4th-degree b-spline interpolation to the same spatial dimensions as the Preop-MRI, which we define as the reference image due to its anatomical integrity. Then, the reference image (i.e., the Preop-MRI) is segmented in its own space into 6 probability tissue maps in both native and MNI IXI549[3] space: grey matter (GM), white matter (WM), cerebrospinal fluid (CSF), bones, muscle and fat, and background map. Then, segmentation is performed based on a mixture of Gaussians model and tissue priors using an extension of the unified segmentation algorithm[4–6]. Basically, parameters for tissue classification, bias correction, and registration onto the space of tissue priors (MNI IXI549[3], by default) are estimated in one single generative model. Therefore, SPM12 segmentation is a comprehensive procedure that also works as a normalization step, yielding results both in the native and tissue priors space using a non-linear registration that minimizes two terms: a measure of similarity (mean-squared difference) between the images and the roughness of the deformations.

Next, the coregistered Postop-MRI is segmented using the probability maps (subject-specific) generated in the previous step as tissue priors. Because of that, this segmentation also creates non-linearly registered maps (GM, WM, and CSF) in the Preop-MRI space.

The non-linearly registered Postop-MRI tissue maps are thresholded (<0.2) to exclude low probability voxels and then binarized. Then, the GM and WM binary maps are summed, smoothed with a small Gaussian filter (full width at half maximum (FWHM) = 3x3x3 mm^3), generating the final postoperative mask. A similar process is performed with the Preop-MRI but without the smoothing step and using a

more stringent threshold (<0.3) to produce the final preoperative mask. Then, the final postoperative mask is subtracted from the final preoperative, smoothed (FWHM = 5x5x5 mm^3), thresholded (<0.1), and binarized, yielding the lacuna segmentation. It is important to highlight that this last smoothing purposely dilates the 3D shape to avoid missing important parts of the lacuna.

As the noise, anatomical variations, pathologies, and even previous invasive procedures may create putative lacunas in the image[1], the algorithm identifies all the non-contiguous clusters (i.e., lacuna candidates) and selects the largest one to be the final result. Due to the choice of the Preop-MRI as the reference image, the final lacuna mask is generated in the Preop-MRI space, with the same field of view and voxel sizes as the Preop-MRI. To bring the lacuna mask into the MNI IXI549 standard space, we use the deformation matrix estimated in the segmentation of the Preop-MRI and apply it to the lacuna mask.

The next step comprises the characterization of the two lacuna masks (one in the standard MNI space and one in the Preop-MRI space). Besides calculating the volume of the entire lacuna, *ResectVol* also quantifies the resected volume and performs the anatomical labeling of the brain structures inside the lacuna. The labeling is performed using the MNI IXI549 template that was segmented using FreeSurfer[7] (version 7.1.1) and the Desikan atlas[8] by identifying the voxels from the lacuna that coincide with regions of the template. The resected portion of these labeled structures is saved as individual masks for each structure, and their volumetric measures are saved in a text file.

If more than one Preop-MRI image from the same subject is used, these multiple inputs are averaged in an attempt to improve the final results because the averaged image takes into account the normal variability related to different imaging protocols and positioning, and also helps to control for preprocessing associated errors. In the case of multiple Postop-MRIs, *ResectVol* treats each image

---

[1] It is worth noting that factors such as noise and artifacts tend to create false lacunas, as opposed to an actual lacuna.

separately, creating independent results since they can be related to distinct postoperative periods with distinct scientific or clinical interests.

Finally, a slice view panel of the surgical lacuna mask overlayed onto the brain-extracted Postop-MRI is saved in the Portable Network Graphics (.png) format to give users a simple but straightforward visualization of the segmentation. By default, images are displayed in axial orientation with 20 slices regularly spaced from the most caudal to the most cranial lacuna boundaries. *ResectVol* supports changes in these parameters (orientation and number of slices), but they must be changed directly in the code since they are not available in the GUI. All steps are schematically organized in Figure S1.

References:

1. Friston KJ, Holmes AP, Worsley KJ, Poline J-P, Frith CD, Frackowiak RSJ. Statistical parametric maps in functional imaging: A general linear approach. Hum Brain Mapp. 1994; 2(4):189–210.
2. Collignon A, Maes F, imaging DD-… in medical, 1995 undefined. Automated multi-modality image registration based on information theory. books.google.com.
3. BIDS-contributors. The Brain Imaging Data Structure (BIDS) Specification (1.6.0). 2021; .
4. The FIL Methods Group and honorary members. SPM12 Release Notes. London; 2014. p. 1–9.
5. Ashburner J, Friston KJ. Unified segmentation. Neuroimage. 2005; 26(3):839–51.
6. Malone IB, Leung KK, Clegg S, Barnes J, Whitwell JL, Ashburner J, et al. Accurate automatic estimation of total intracranial volume: A nuisance variable with less nuisance. Neuroimage. 2015; 104:366.
7. Fischl B. FreeSurfer. Neuroimage. 2012; 62(2):774–81.
8. Desikan RS, Ségonne F, Fischl B, Quinn BT, Dickerson BC, Blacker D, et al. An automated labeling system for subdividing the human cerebral cortex on MRI scans into gyral based regions of interest. Neuroimage. 2006; 31(3):968–80.
