# Supplementary material for: ResectVol: A tool to automatically segment and characterize lacunas in brain images
Source: Epilepsia Open. 2021 Oct 12;6(4):720–6. doi: 10.1002/epi4.12546 (PMC8633465; doi:10.1002/epi4.12546)
Supplement: Supplementary file 4 — Supplementary Material [file EPI4-6-720-s002.docx]

# Supporting Information

***Figure S1. Processing pipeline.*** *Illustration of the processing steps of the MR images to create the final lacuna mask. Preop-MRI: preoperative MRI; Postop-MRI: postoperative MRI; GM: gray matter; WM: white matter.*

***Appendix S1.*** *ResectVol development.*

***Table S1.*** *Imaging parameters.*
